# Characterization of nZVI nanoparticles functionalized by EDTA and dipicolinic acid: a comparative study of metal ion removal from aqueous solutions

**DOI:** 10.1039/c9ra04831f

**Published:** 2019-10-01

**Authors:** Sanda Rončević, Ivan Nemet, Tea Zubin Ferri, Dubravka Matković-Čalogović

**Affiliations:** Department of Chemistry, Faculty of Science, University of Zagreb Horvatovac 102a 10000 Zagreb Croatia roncevic@chem.pmf.hr +385 1 4606 181 +385 1 4606 182; METRIS Materials' Research Centre Zagrebačka 30 52100 Pula Croatia

## Abstract

The simultaneous adsorption of metal ions on bare and functionalized zero-valent iron nanoparticles (nZVI) from aqueous solution was tested using inductively coupled plasma optical emission spectrometry (ICP-OES). The nanomaterials were synthetized using borohydride reduction of iron salt followed by addition of EDTA and pyridine-2,6-dicarboxylic acid (dipicolinic acid, PDCA) in different molar ratios. Functionalized materials were characterized by FT-IR, XRD and SEM-EDS methods. The ligand attachment on the particles was confirmed by FT-IR spectroscopy. The formation of a magnetite and feroxyhyte shell on the core of functionalized nanoparticles was confirmed by the XRD study. Transformation of chain-like structures into clusters of nanospheres with smaller diameter size was observed from SEM study of EDTA-nVZI particles. The average diameter of bare nZVI particles comprised 115 nm, while EDTA functionalization resulted in an average diameter of 22 and 35 nm. The PDCA-nZVI particles obtained with the molar ratio of Fe : PDCA = 1 : 1 retain the chain-like structure with enlargement of the average particle diameter to 267 nm. SEM study of PDCA-nZVI particles that were produced using the ratio Fe : PDCA = 2 : 1 have demonstrated the unique property of elongation into ellipsoidal forms of reduced dimensions (*a* = 61 nm; *b* = 28 nm). The simultaneous metal ion removal from aqueous solution was the most efficient in the case of bare nZVI particles (91–97%). EDTA functionalization was found to be highly selective for Cu and Cr removal (95%), while PDCA functionalization shows selective adsorption of Cu, Cr and V in an aqueous medium (93%). Iron nanoparticles functionalized with PDCA in both of the used ratios showed more efficient metal ion adsorption in the case when smaller ellipsoidal particles were formed.

## Abstract 2

Surface modification of nZVI particles by EDTA and PDCA leads to the formation of magnetite and feroxyhyte shell. PDCA capping caused the elongation of spheres into ellipsoids. Metals adsorption was more efficient on smaller ellipsoidal particles.

## Graphical abstract

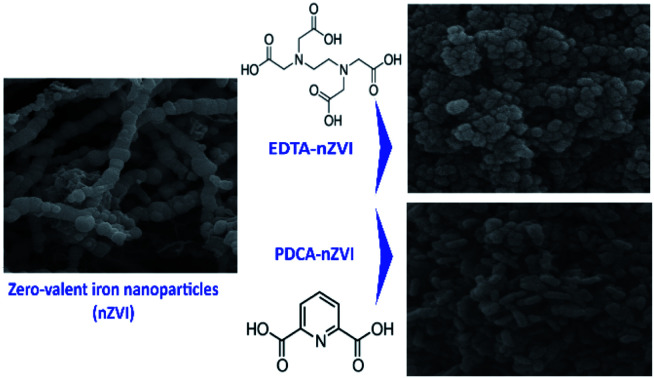

## Introduction

Special physical and chemical properties of engineered nanoparticles have directed research interest towards production and use of new materials in many different fields of application. Over the past decade, considerable great attention has been paid to utilization of iron-based nanoparticles. Generally, they are prepared as supermagnetic iron oxide nanoparticles (SPION) and zero-valent iron nanoparticles (nZVI).^1^ The first group of iron-based nanomaterials have shown exclusive potential in biomedical applications such as drug delivery agents or contrasting agents for magnetic resonance imaging,^2–4^ while the second group is widely applied in environmental remediation and water cleaning technology.^5–8^ Zero-valent iron nanoparticles (nZVI) demonstrated a great potential in removal of specific hazardous substances, such as heavy metals^9–13^ and organic pollutants from water and soil systems.^14,15^ It is due to the exceptional properties of cost-effective nZVI particles, *i.e.* large surface area and high reactivity. These properties are reflected through more efficient adsorption and reduction mechanisms on core–shell structures of nZVI, when compared to iron powder of standard micrometer size.^16^ However, the oxidation or corrosion of nZVI material in oxic and even in anoxic conditions might inhibit these mechanisms by transforming metal iron to oxides and/or hydroxides and consequently decrease their reactivity.^17^ Furthermore, the strong agglomeration potential and rapid sedimentation complicate their handling and hinder their applicability in remediation purposes.^18^ In order to improve their applicability, various methods of surface modification of nZVI have been studied. Enhancements of colloidal stability and better control of their reactivity are often accomplished by chemical functionalization of surface. Different strategies of nZVI surface alteration are elaborated in the literature. They are generally directed to the selection of suitable carriers such as surfactants, polymers or inorganic materials;^19–24^ and/or to the selection of suitable capping agents for coating of the particle surface.^25–30^ Recently, some superior properties in adsorption processes were recorded using novel materials that consist of iron-nanoparticles metal–organic frameworks (MOFs).^31,32^

Functionalization of nZVI materials is often characterized by more than one analytical technique, such as electron microscopy, X-ray techniques and light scattering. Although the modified nZVI particles are particularly suitable for applications in various analytical chemistry studies, *i.e.* sample preparation, separation and sample preconcentration, the crucial methods in analytical procedure control rely on quantitative spectrometry.^33,34^ For this reason, atomic absorption spectrometry (AAS), plasma optical or mass spectrometry (ICP-OES, ICP-MS) along with UV/VIS spectrophotometry are widely applied in evaluation of nZVI effects on analytical sample preparation procedure.^12,35–37^ In addition, the usability of bare and surface modified nZVI as a potential remedy for minimization of interference effects in atomic spectrometry, is also in focus of scientific researches.^38^

Metal-binding organic ligands such as ethylenediamine tetraacetic acid (EDTA) and 2,6-pyridinedicarboxylic acid or dipicolinic acid (PDCA) are widely used as capping or extractive reagents for transition and rare earth metals. Functionalization of cross-linked polymer networks and maghemite particles by EDTA were studied recently.^39,40^ Reported improvements denote to removal of heavy metals such as Pb, Cd, Cu and As from natural and wastewaters.^41^ The changes on the surface of EDTA functionalized iron oxide nanoparticles were studied by Magdalena *et al.*, who noticed the reduction of particle size after ligand attachment.^42^ The reduction of size that changed the distribution of charges on the surface, they pointed out as a promising result for removal of heavy metals. Contrasting to remediation applications of EDTA containing nanomaterial, PDCA is mostly examined as coordination ligand in luminescent complexes of lanthanides.^43^ For example; functionalization of clay minerals with PDCA produced a hybrid organic–inorganic material that was studied as a potentially luminescent material in Eu and Tb complexation.^44^ Thus, luminescent nanohybrids such as dipicolinate complexes of lanthanides embedded into silica nanoparticles are investigated for potential bioimaging applications.^45^ Surface modification by PDCA was studied on the example of silver nanoparticles, which served as a sensitive colorimetric sensor for detection of traces of bismuth in waters.^46^ However, there is a lack of published data on utilization of PDCA as a capping reagent for nZVI particles.

The functionalization of nZVI surface by the EDTA or PDCA ligand can affect the adsorption of metals. In the light of atomic spectrometry measurements, such property is beneficial in terms of minimization of matrix interferences of complex samples by efficient removal of the interfering metal. Therefore, in this work, we synthesized bare and functionalized nZVI particles and tested their removal efficiency of selected metals from model solutions by the ICP-OES method. Zero-valent iron nanoparticles (nZVI) were synthesized by the method of ferric iron reduction using sodium borohydride as a reducing agent and they were subsequently functionalized by addition of EDTA and PDCA reagents. A systematic characterization of the prepared bare and coated particles was performed using infrared spectroscopy, XRD and SEM methods are also presented in this study. The characterization methods confirmed the ligand attachment on iron particles. We observed the particle size variation that depends on the kind of capping reagent. Moreover, by changing the molar ratio of the starting reagents during the preparation of PDCA-nZVI particles, some interesting changes of particle shape were found. The spherical shape of particles was disturbed and ellipsoidal elongation occurred. To the best of our knowledge, this is the first report of such phenomenon obtained after nZVI functionalization with dipicolinic acid. The prepared bare and functionalized nZVI particles were tested as an adsorptive material for Cd, Co, Cu, Cr, Mn, Ni, and V when present as a mixture of metal ions in an aqueous solution. The metal ions removal efficiency of the prepared EDTA-nZVI and PDCA-nZVI particles was compared to ion removals of bare nZVI particles and the observed effects are discussed in this work.

## Experimental

### Synthesis and functionalization of nZVI particles

Synthesis of nZVI particles was performed by sodium borohydride reduction as described by Whang and Zhang^16,47^ and modified according to Ruiz-Torres *et al.* by using an open vessel at regular atmospheric conditions.^30^ The starting solution was prepared by dissolution of 1 g of FeCl_3_·6H_2_O in 100 mL of deionized distilled water in a 400 mL beaker. An amount of 0.70 g of NaBH_4_ was dissolved in 100 mL of water. Freshly prepared solution of NaBH_4_ was added into the iron chloride solution under vigorous stirring. The black magnetic precipitate was filtered by vacuum filtration and washed with deionized distilled water. Prepared bare nZVI particles were stored in containers under ethanol to prevent further oxidation.

Functionalized particles were prepared in the same manner but with addition of EDTA or PDCA ligands to the starting iron chloride solution. An amount of 1.3771 g of EDTA that was needed for the molar ratio of Fe : EDTA = 1 : 1, was dissolved in 100 mL of water and added into the iron containing solution. The borohydride reduction was performed afterward and the EDTA containing precipitate was filtered, washed and stored under ethanol. The obtained product was non-magnetic brown-orange precipitate with no black nanoparticle appearance. In the further experiments molar ratios of 2 : 1, 3 : 1 and 4 : 1 were used. The black coloured magnetic nanoparticles were obtained in the last two experiments, *i.e.* Fe : EDTA = 3 : 1, and Fe : EDTA = 4 : 1. These products were used for the further characterization and sorption experiments.

Solution of dipicolinic acid (PDCA) was prepared by dissolution of 0.7112 g of pure compound in 150 mL of deionized distilled water. Due to the poor solubility of PDCA, the solution was prepared by use of ultrasonic bath. Solution was placed into beaker containing dissolved iron chloride and the reduction process was performed by addition of NaBH_4_ during stirring. The black precipitate of magnetic nanoparticles formed instantaneously after addition of the reducing reagent. The PDCA-nZVI particles obtained by using the molar ratio Fe : PDCA = 1 : 1 were filtered, re-flushed with water, and stored under ethanol. The experiment was repeated with the ratio Fe : PDCA = 2 : 1 and black coloured magnetic particles were formed.

### Solid-phase characterization of functionalized particles

Morphology of the obtained particles was observed by scanning electron microscopy (SEM) on a Thermo Fisher Scientific FEI Quanta 250 FEG. Particle images were obtained operating in high vacuum mode (10^−4^ Pa to 30^−3^ Pa) on Au–Pt coated samples using secondary electrons detector (Everhart Thornley Detector, ETD) and acceleration voltage of 7 to 10 kV. Crystalline phases were determined by powder X-ray diffraction on a Philips X'Pert diffractometer (2*θ* range 15–100°) in the Bragg–Brentano geometry using Cu Kα radiation. The samples were placed on Si sample holders. Patterns were visualized using the X'Pert program HighScore.^48^ Infrared spectra were obtained from KBr pellets using FTIR Bruker Vector 22 in the wave range of 4000–450 cm^−1^ (50 scans acquisition, resolution 4 cm^−1^).

### Experiments in solution

The ICP-OES analyses of solutions were performed with a Teledyne Leeman Labs. Prodigy High Dispersion ICP. The instrument is equipped with 40 MHZ “free-running” radiofrequency generator and echelle grating spectrometer with a large-format programmable array detector (L-PAD). Sample introduction system consists of a peristaltic pump, glass cyclonic spray chamber and a glass concentric nebulizer. The dual-view torch for observing both axial and radial position was used. The solution uptake rate was adjusted on 1 mL min^−1^. The radiofrequency power of 1.2 kW, and flow rates of argon (coolant 18 L min^−1^, auxiliary 0.9 L min^−1^) were held constant during measurements. Emission lines of elements were selected from image on L-PAD detector as the best ones, *i.e.* lines without spectral and background interferences. Single element standard solutions of Cd, Cr, Co, Cu, Fe, Ni, V, and Zn (Plasma Pure, Leeman Labs, Hudson, NH, USA) and multielement standard ICP-Mehrelement-Standardlösung IV (Merck, Darmstadt, Germany) were used for the control of plasma positioning and preparation of calibration standard solutions. All calibration standards were prepared by appropriate dilution of standard stock solutions (1 g L^−1^) in the concentration range from 0.1 to 100 mg L^−1^. Nitric acid was added in each calibration solution up to the final concentration of 2% v/v.

Stock multielement standard solution of 100 mg L^−1^ of Cd, Cr, Co, Cu, Fe, Ni, V, and Zn, was used in the sorption experiments. An amount of 0.1 g of synthetized particles was placed into conical flask where the volume of 100 mL of multielement solutions was added. The final concentration of metals in the flask was 10 mg L^−1^. Flasks were sonicated for 30 min in an ultrasonic bath. Filtration of supernatant was performed using SPE manifold on cartridges with silica gel beds. Metal concentrations were measured directly from filtered solutions by the ICP-OES method. Nanoparticles that remained on cartridges after filtration were dissolved by addition of 5 mL of concentrated nitric acid. The obtained solutions were diluted to 25 mL with distilled deionized water. Concentration of metals in these solutions denotes adsorption on nZVI particles and it was determined by the ICP-OES method. The procedure was repeated separately for each kind of synthesized nanoparticles, *i.e.* bare nZVI and functionalized nZVI particles.

## Results and discussion

### Characterization of bare and functionalized nZVI particles

#### FT-IR characterization

**Fig. 1:**
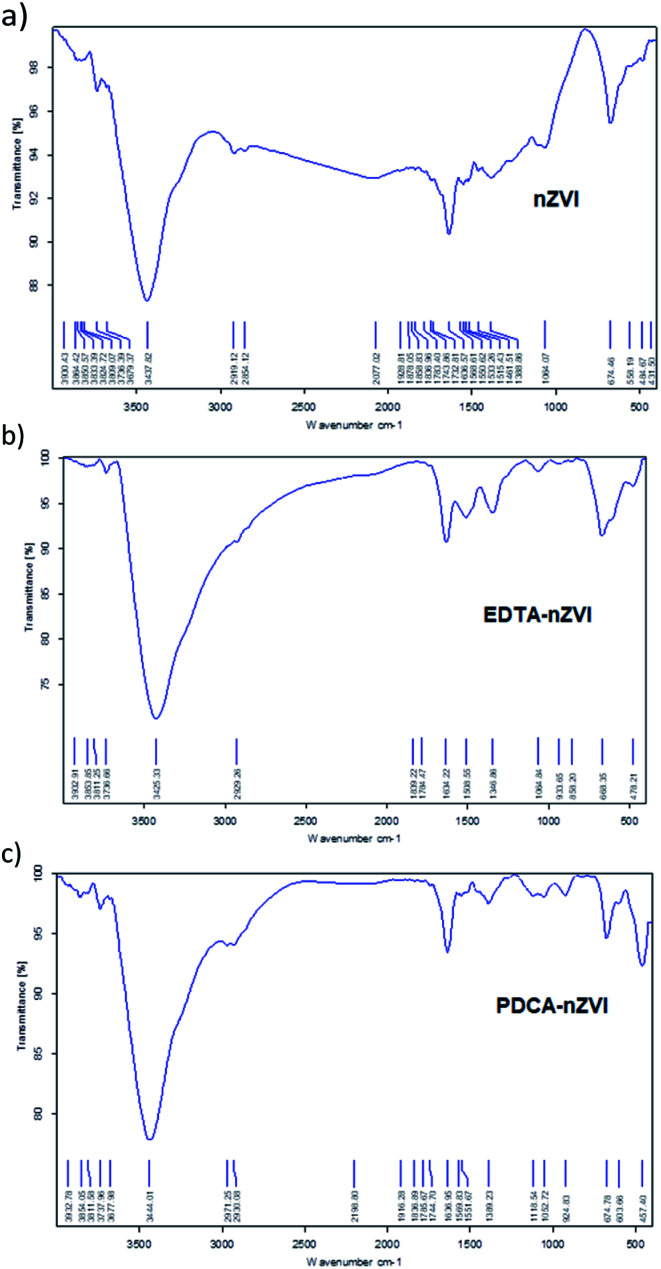
FT-IR spectra obtained in KBr pellets for: (a) bare nZVI particles, (b) EDTA functionalized nZVI particles, (c) PDCA functionalized nZVI particles.

Chemical bonds that occur during functionalization of nZVI particles were examined by FTIR spectroscopy and resulting spectra are showed in Fig. 1. Infrared spectrum of bare nZVI particles presents: a band at 3437 cm^−1^ that denotes to O–H stretching vibration; a band at 1636 cm^−1^ that denotes to O–H bending; and a band at 674 cm^−1^ that denotes to stretching vibration of Fe–O. Functionalization with EDTA shows appearance of additional vibration bands at 1389 cm^−1^ that denotes to out-of-plane bending of CH_2,_ and a band at 1061 cm^−1^ that denotes to stretching of C–N. The vibrational bands of O–H bending and Fe–O stretching at 1624 cm^−1^ and 583 cm^−1^, respectively, are more pronounced in EDTA-nZVI than in the spectrum of bare nZVI particles. The FTIR spectra of products with molar ratios Fe : EDTA = 3 : 1 and Fe : EDTA = 4 : 1 are generally similar and there are no evidence of significant shifts in absorption bands. Changes in magnitude of vibrational frequencies compared to pure ligand absorption were discussed in the several studies.^30,40,41^ The authors attributed the attenuation of intensity of characteristic ligand vibration to interaction of O atoms of ligand with the Fe atoms through coordinated covalent bonds. The similar results of our study confirm the attachment of EDTA ligand on surface of nZVI particles.

In the spectrum of PDCA-nZVI particles, the appearance of aromatic C–C fundamental vibration is visible at 1052 cm^−1^ along with an overtone vibration near 2061 cm^−1^. Besides O–H vibration at 3444 cm^−1^, there are also present bending and stretching vibrations of O–H and Fe–O at 1636 cm^−1^ and 674 cm^−1^, respectively. The additional Fe–O vibration at 457 cm^−1^ becomes visible in the product of molar ratio Fe : PDCA = 2 : 1, while in the product of molar ratio Fe : PDCA = 1 : 1 is missing. The similar appearance of two bands at the lower wavenumber range was established in the conditions of green synthesis.^49^ The authors investigated the nZVI synthesis using green tea extracts and they attributed Fe–O vibrations in FTIR spectra to the formation of Fe_2_O_3_ and Fe_3_O_4_ on the surface of particles. The appearance of similar vibrational bands is observed in PDCA-nZVI spectra, which suggests the capping of ligand on iron particles surface.

#### XRD characterization

**Fig. 2:**
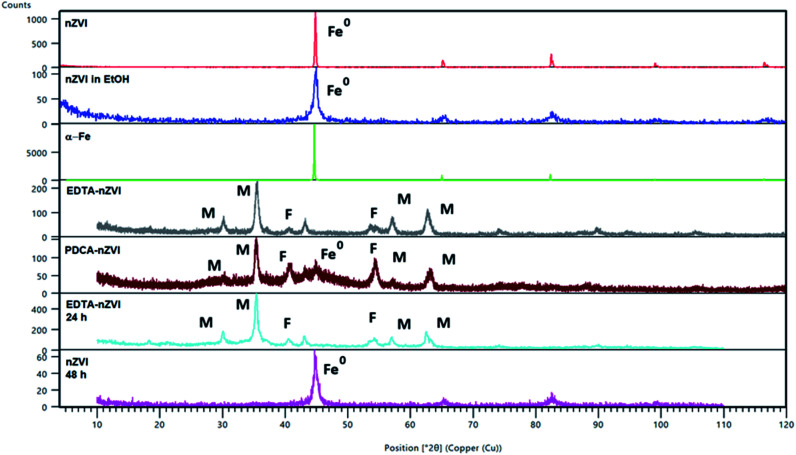
PXRD diffraction patterns of α-Fe and of bare and functionalized nZVI particles. Peaks are referred to magnetite (Fe_3_O_4_) (M), feroxyhyte (δ-FeOOH) (F) and nZVI (Fe^0^). Two patterns were calculated from the structures of magnetite (26410-ICSD)^50^ and feroxyhyte (38299-ICSD).^49^

The XRD patterns of bare and functionalized nanoparticles are presented in Fig. 2. Diffraction maximum at 2*θ* = 45° represents the characteristic peak of zero-valent iron (α-Fe). Diffraction patterns of dried non-functionalized nZVI particles and particles in an ethanolic suspension are almost identical and correspond to that of α-Fe. There is no evidence of oxidation reactions on non-functionalized nanoparticles.

After functionalization of nZVI particles with EDTA, the diffraction pattern shows appearance of magnetite (Fe_3_O_4_). Two additional weak maxima at 2*θ* of 40° and 54° indicated a smaller amount of an additional phase which was characterized as feroxyhyte (δ-FeOOH). This is the first example of this mineral observed on nZVI particles. Feroxyhyte is a poorly crystallized material and the XRPD patterns reported in the literature show a diffuse background with a broad band at low angles and four discrete but broad maxima.^50,51^ The composition of this oxyhydroxide is not well defined as a result of hydrolysis at the nanometric granule surfaces and of possible presence of multiple phases. The XRD patterns of the natural and synthetic δ-FeOOH show differences depending on the materials origin or preparation route.^50^ There are three structural models for the structure, the best correlation with the maxima found in our powder patterns is that of Patrat *et al.*^51^

In contrast to the EDTA-nZVI pattern, in the diffraction pattern of PDCA functionalized particles we can observe the remaining peak of α-Fe. The PDCA-nZVI diffractogram also shows the characteristic maxima of magnetite but it is present in a smaller quantity then in EDTA-nZVI.^52^ However, there is much more feroxyhyte and there is also a significant amount of an amorphous phase. In the several published studies that denote modification of iron nanoparticles with dithizone, EDTA and ethylene glycol, a similar pattern of formed magnetite was observed.^27,30,42^ The transformations due to oxidation on particle surface are complex. The appearance of additional maxima is generally explained by oxidation mechanisms. Maghemite (γ-Fe_2_O_3_) and iron oxyhydroxide (FeOOH) are the most common additional phases in several similar studies.^17,42^ The recent study from Liu *et al.* considers oxidation mechanisms of nZVI in water and explains the conditions under which some particular oxides are formed.^17^

Under anoxic conditions, the oxidation products contained a mixture of wustite (FeO), goethite (α-FeOOH) and akaganeite (β-FeOOH), while under oxic conditions, the final product was mainly lepidocrocite (γ-FeOOH). In our XRD patterns no maxima corresponding to any other FeOOH phase apart from δ-FeOOH were found, nor was any Fe_2_O_3_ phase present. We found no visible difference in diffraction patterns by changing the Fe/ligand ratio with both of the examined ligands.

#### SEM characterization

**Fig. 3:**
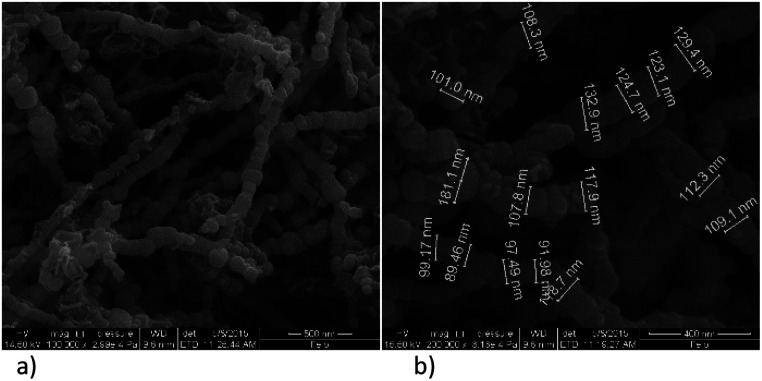
SEM images of bare nZVI particles; (a) nanochains, (b) diameter of spheres under magnification of 200 000×.

SEM images of synthetized nanoparticles without ligand addition display dendritic forms that are build-up of nanochains (Fig. 3). The diameters of spherical individual particles are not uniformed and they vary from 90–180 nm with the average diameter of 115 nm. The chain-like structure in non-functionalized nZVI material is caused by magnetic dipole–dipole interactions, which leads to adherence of spherical particles and their arrangement.^30^

**Fig. 4:**
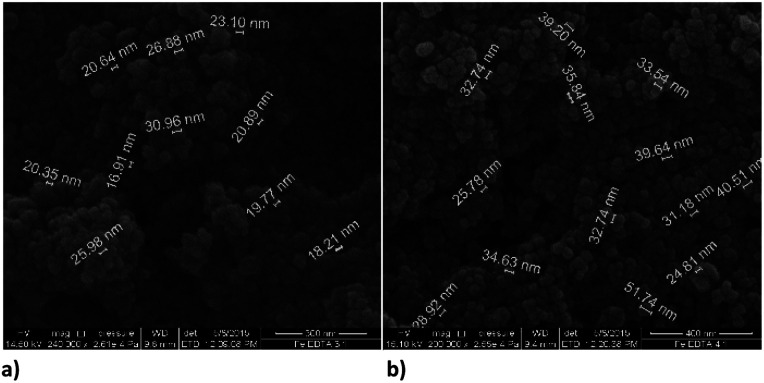
SEM images of EDTA functionalized nZVI particles; (a) molar ratio Fe : EDTA = 3 : 1 (b) molar ratio Fe : EDTA = 4 : 1.

Functionalization with EDTA cause reduction of the particles size, which is evident from the SEM images, Fig. 4, and it was confirmed in both of the observed ratios, *i.e.* Fe : EDTA = 3 : 1 and Fe : EDTA = 4 : 1. The average diameter of spheres obtained with the first ratio was 22 nm, and the second ratio shows 35 nm. The dendritic structure of nanochains is not evident as was with the bare nZVI particles. The grouping in clusters or cumulated piles of spherical particles occurred. The similar behaviour was examined in the study of Magdalena *et al.* who found that EDTA acts as a barrier to the growth of iron oxide nanoparticles and thus the size of particles decreased.^42^ Their TEM study showed a highly homogeneous shape and size of EDTA functionalized particles. Studies of functionalization of magnetic oxide nanoparticles that are immobilized on different carriers shows the same effect of EDTA capping.^39–41^ The formation of similar homogeneous material is observed here with the starting material consisting of nZVI instead of commercial magnetite nanoparticles.

**Fig. 5:**
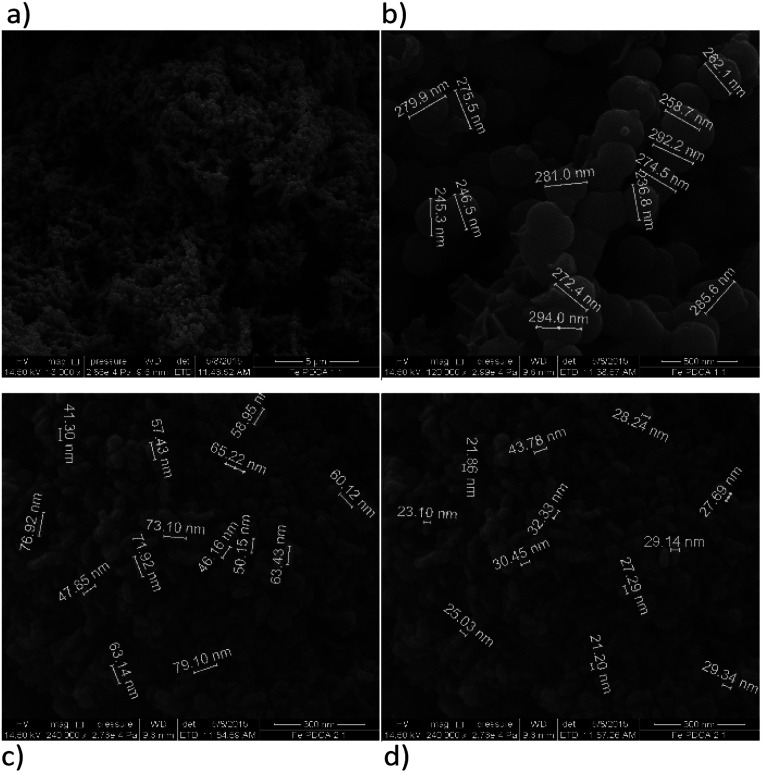
SEM images of PDCA functionalized nZVI particles; (a) short nanochains (b) diameter of spheres with molar ratio Fe : PDCA = 1 : 1, (c) and (d) ellipsoidal particles with molar ratio Fe : PDCA = 2 : 1.

Functionalization of nZVI with PDCA observed by SEM shows differences in the shape and size of particles, which depends on the Fe/ligand ratio employed into synthesis (Fig. 5). The particle size of spheres after functionalization in the ratio Fe : PDCA = 1 : 1 comprise average diameter of 267 nm. The spheres comprise more than a double size of bare nZVI. Comparing to bare nZVI particles, morphology of the PDCA functionalized material changed into short chain structures. A surprising phenomenon is observed when the Fe/ligand ratio during synthesis was changed to Fe : PDCA = 2 : 1. The SEM image shows the reduction of particle size, and unexpectedly, the shape of particles changed through elongation of particles in one dimension. The alteration of spherical particle shape to ellipsoidal forms was observed. The average length of the oblong particles is 61 nm and the average width comprises 28 nm. The short-chain structures in morphology are missing and the forming piles of particles resemble those with EDTA capping.

From the literature survey, there are no data of similar observations which refer to PDCA capping of zero-valent iron or iron oxide nanoparticles. The sparsely published research mostly deals with the use of PDCA in lanthanide ions sensing, especially after its incorporation in silica matrix.^42–45^ For this reason, the understanding of iron-dipicolinic acid complex formation in solutions might be helpful for possible explanation of shape alteration, as we observed here. The unique structural considerations of iron complexes with the PDCA ligand can be found in series of papers published by Laine, Gourdon *et al.*^53–56^ They have examined mononuclear and polynuclear complexes of iron(iii) and iron(ii) that they isolated from acidic solutions of different pH. Their studies revealed variations of bond length that were induced by the change of the iron oxidation state. Thus, the carboxylate group bonding through Fe–O increased with addition of one electron to the iron(iii) complex.^53^ Laine *et al.*, have found rather complex structural consequences, and for this reason, they were not able to correlate solution chemistry and solid state structures.^54^ The latest published research takes into consideration theoretical calculations on the iron(ii)-dipicolinic acid complexes.^57^ Authors applied quantum theory of atoms and molecules (QTAIM) to characterize the nature of chemical bonds in the corresponding complexes. They have confirmed the elongation of C–O bonds in the examined complexes, but moreover, they found the magnification of ellipticity values for the Fe–O and Fe–N bonds that affects the geometrical parameters of molecules.

**Fig. 6:**
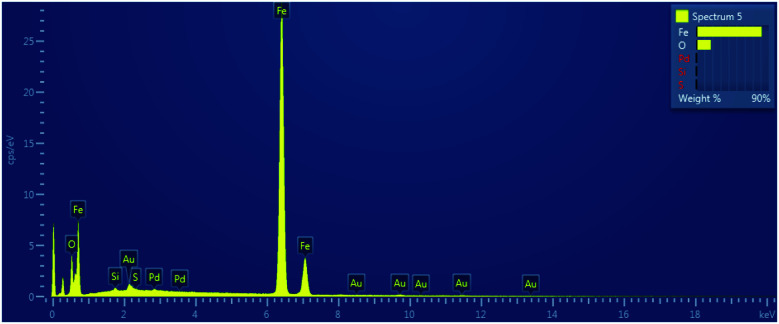
SEM-EDS mapping of nZVI; the example of Fe : PDCA = 1 : 1 nanoparticles.

In order to get the insight into composition of prepared nanomaterials, the SEM-EDS mapping was performed and the example of obtained scan is presented in Fig. 6.

The measured mass fractions of main elements Fe and O for each of examined type of material that were estimated after SEM-EDS elemental mapping are presented in the Table 1.

**Table 1:** Mass fractions of iron and oxygen in synthesized nanomaterials estimated by SEM-EDS method

Type of synthesized particle	*w*(Fe)/%	*w*(O)/%
Bare nZVI	90.45	7.96
EDTA-nZVI [Fe : EDTA, 3 : 1]	71.67	25.43
EDTA-nZVI [Fe : EDTA, 4 : 1]	57.95	37.86
PDCA-nZVI [Fe : PDCA, 1 : 1]	77.55	20.70
PDCA-nZVI [Fe : PDCA, 2 : 1]	43.85	49.60

The functionalization with EDTA and PDCA changes the mass percentage of oxygen in the systems. Thus, from the Fe/EDTA ratio of 3 : 1 and the Fe/PDCA ratio of 1 : 1 is evident that the values of mass fraction are the closest to the theoretical fraction values of magnetite, *i.e. w*(Fe) = 72.4% and *w*(O) = 27.6%. For comparison purposes, the theoretical mass fraction values of feroxyhyte are *w*(Fe) = 63% and *w*(O) = 36%. It is also interesting that lower starting ligand concentration results in a higher oxygen content in the examined nanoparticles. This is related to the formation of iron oxyhydroxide, (FeOOH) on the particle surface,^58^ in our case δ-FeOOH. For the explanation of observed distribution, the future investigations should involve some additional methods. For example, XPS or XANES can help to obtain better insight into iron(ii) and iron(iii) equilibria on the core of prepared particles.

#### Determination of metals removal from aqueous solution by ICP-OES

Analytical performances of inductively plasma optical emission spectrometry (ICP-OES) provide sensitive simultaneous measurements of different emission lines in solutions of complex matrix and therefore, it was applied in our study of metal removal from aqueous solutions by nZVI. Multielement standard solution containing Cd, Cr, Co, Cu, Ni, V, and Zn, was used in the experiments that are performed using bare and functionalized nZVI. It is already known that sample handling of nanoparticle materials and preparation of suitable solutions for introduction into plasma are the most critical steps in measurement procedure. Direct sampling is generally favoured approach in plasma spectrometry, but the broad particle size distributions in nanomaterial suspensions make them unsuitable for direct introduction into plasma source. Another method of sample introduction is slurry sampling of suspensions, but it requires the particle size range of 5–20 nm.^34^ Problems that arise from slurry introduction into plasma become critical when the particle sizes exceed 20 nm.^35^ Therefore, dissolution of solid phase by digestion of particles using mineral acids is often applied.^38^

In our work, the removal of selected metals was tested first by measuring the concentrations in filtrate collected after extraction on SPE cartridges. After separation, the remaining solid material was dissolved by addition of nitric acid and adsorbed metal concentrations were determined. The compromise conditions of ICP-OES parameters were chosen for all multielemental determinations. However, in solutions of dissolved nanoparticles some effects on measuring signals are expected.^59^ Spectral overlapping or matrix interferences may occur due to the high iron content in digested samples. In order to prevent and to minimize these effects, several preliminary procedures were employed. The first was background subtraction that was performed on L-PAD detector of ICP-OES instrument by acquiring of total iron spectrum. The emission spectrum was subtracted subsequently from total emission line spectra of prepared multielement solution. The most prominent lines of elements, which were free from iron interferences, were selected for the measurements. The matrix-matching of calibration solutions was also performed. For this purpose, the standard iron solution was added in all multielement calibration standards to final concentration of 100 μg L^−1^. The linearity of curves on all measured lines comprise the satisfactory *R*^2^ coefficient values (0.9989–0.9999) along with measurement precision of 0.1–2.0% RSD. The detection limits of elements in high iron load matrix that were obtained after adjustment of measured signals are shown in Table 2.

**Table 2:** Element lines and limits of detection in high iron load matrix

Element	Emission line *λ*/nm	Limit of detection (LOD) μg L^−1^
V	292.401	1.2
Cr	357.868	4.0
Mn	257.610	0.1
Co	228.615	5.0
Ni	231.604	1.0
Cd	228.802	0.1
Zn	213.857	1.0
Fe	259.940	0.2
Cu	224.700	0.5

**Fig. 7:**
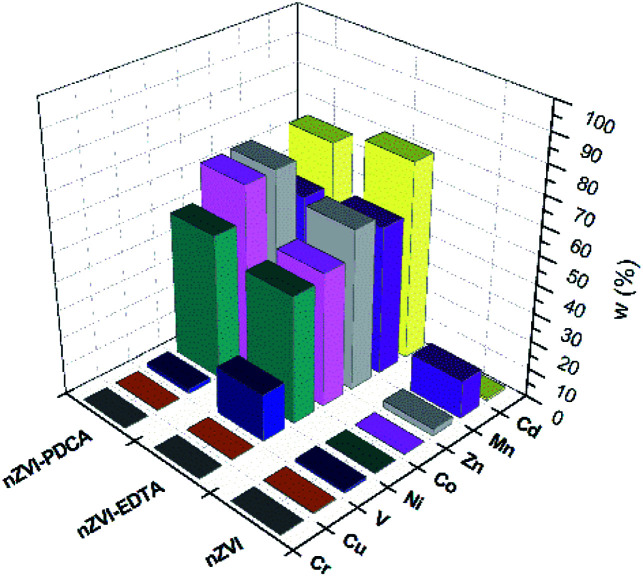
Metal concentrations in filtrate solution after adsorption on bare, EDTA and PDCA functionalized nZVI particles measured by ICP-OES method.

The measured concentration of metals in supernatant solution after adsorption on bare nZVI particles, EDTA-nZVI, and PDCA-nZVI are shown graphically in Fig. 7. Concentrations are presented as a percentage ratio of measured value to added standard of 10 ppm. It is visible that elements content in filtrate after adsorption on bare nZVI particles is significantly lower than in experiments with functionalized particles. The exceptions are Cr, Cu and V in sorption experiments performed with EDTA and PDCA functionalized particles.

**Fig. 8:**
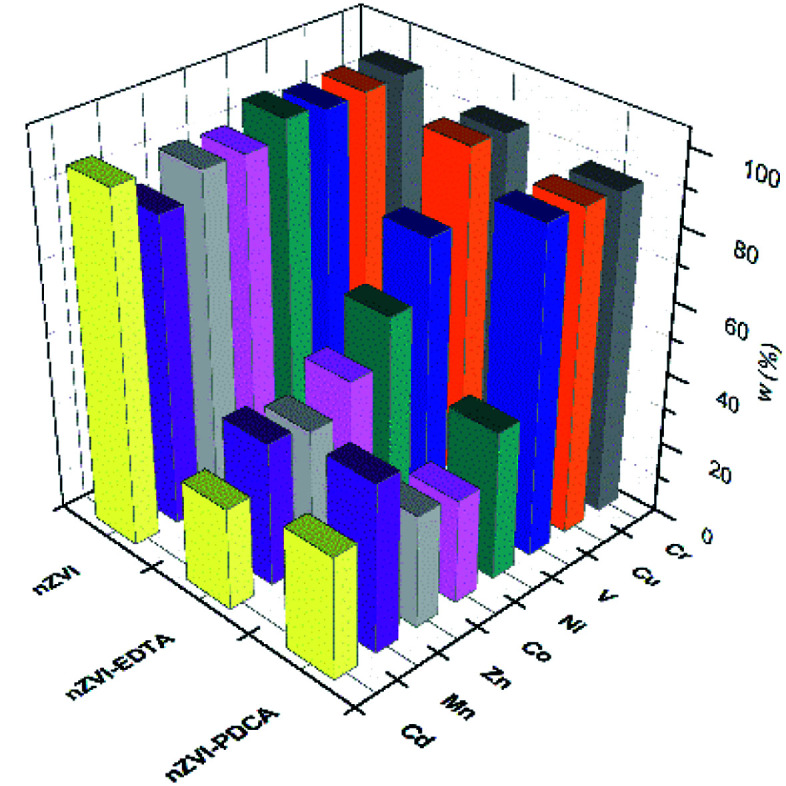
Adsorbed metal concentrations on bare, EDTA and PDCA functionalized nZVI particles measured by ICP-OES method after dissolution of solid phase.

Dissolution of nanomaterials after adsorption experiments with mutilelement solution gives the concentration values that are shown in Fig. 8.

The most efficient adsorption of all examined metals is observed in the case of bare nZVI particles used as adsorbent. The efficiency of metals removal from aqueous solution takes 91–97% of starting mass, with the exception of manganese (85%). By superposition of metal content, which was measured in supernatant and in dissolved nanoparticles, the overall concentration was lower than starting standard concentration. Deviations comprise 3–9% lower values than reference value of 10 ppm. The exception was the sum of Mn concentrations, which exceed the reference concentration for 2.5%. This is the most likely due to the interference effect from neighbouring ionic line of Co.^60^ Experiments with bare nZVI particles confirm simultaneous removal of different metals from aqueous solution.^13^

Functionalization of particles with EDTA in ratio Fe : EDTA = 3 : 1 reveals that high efficiency of removal denotes only to Cr, Cu and V (95%), while the other elements were adsorbed in lower extent (28–46%). The experiments with synthetized particles with ratio Fe : EDTA = 4 : 1 do not show significant changes in measured metal concentrations. The overall results comprise the same range and the most similar values as was measured with the particles of ratio Fe : EDTA = 3 : 1. This observation might be attributed to the similarity of both kinds of synthesized material, which is confirmed by XRD and SEM studies.

The removal mechanisms of bare nZVI particles for different heavy metals were widely studied and they could vary depending on the standard reduction potential of metals.^9,13,61^ Metal removal from aqueous solutions could be described through sorption, reduction and precipitation. The combination of these processes is also possible due to dual properties of core–shell structure of iron particles.^16,61^ However, ligand attachments on nZVI particles cause changes of removal mechanism towards adsorption and complexation.^27,28,40^ The oxide layer on particle provides reactive sites for coordinative reactions.^10^ Both types of interaction are susceptible on changes of pH and competitive adsorption or complexation of other cations in solution.^8,40^ For this reason, the removal efficiency of EDTA-nZVI particles is slightly degraded in multielemental solution when compares to bare nZVI. The highest removal of Cu and Cr among other metal ions implies the strongest complexation on EDTA-nZVI particles. Functionalization with PDCA resemble the adsorption behaviour with EDTA that was demonstrated above. Adsorption efficiency of Cu and Cr takes 92% and 93%, respectively. The exception is noted in a weaker adsorption of vanadium (73%) when compared to the EDTA-nZVI material. In addition, the slightly favoured adsorption with PDCA is observed on the measured Co and Ni concentrations. This might be attributed to stronger complexation of Co and Ni on PDCA-nZVI surface in comparison to other metal ions. For the more detailed insight in prevailing mechanism, the effects of changes of pH is planned for the further extended study on metal ions removal efficiency of functionalized nZVI particles.

The rise in concentrations of all adsorbed metals were noted in experiments with particles of the ratio Fe : PDCA = 2 : 1 when compared to the experiments with particles of the ratio Fe : PDCA = 1 : 1. This enhancement denotes 5% of element concentrations that were measured in the Fe : PDCA = 1 : 1 nanomaterial. The observed change can be related to alteration of the particle size and shape which is confirmed by the SEM study. The smaller ellipsoidal particles of the material obtained with ratio Fe : PDCA = 2 : 1 obviously have a larger active surface and consequently the adsorption is more efficient.

## Conclusions

This work demonstrated the removal of metal ions from aqueous solutions using bare and functionalized nZVI particles, which was analysed by ICP-OES method. Functionalization of particles was performed during synthesis of nZVI by addition of EDTA or PDCA in different molar ratios. The synthesized nanomaterials were characterized by FT-IR, XRD and SEM-EDS methods. Feroxyhyte (δ-FeOOH) was for the first time found on the functionalized nanoparticles by XRD. Reduction of particles size and alteration of chain-like structures of nZVI were observed after functionalization with EDTA. Particles formed by the ratio Fe : PDCA = 1 : 1 showed increase in size, while those prepared by ratio Fe : PDCA = 2 : 1 showed an alteration of spherical nanoparticles shape. The unique feature that we observed in this experiment is an elongation of particles in ellipsoidal forms of reduced size. The modification of particle surface affects the adsorption capabilities of the nZVI particles. This was tested using the ICP-OES method on Cd, Cr, Co, Cu, Ni, V, and Zn removal from aqueous solutions. The bare nZVI particles showed the most efficient and simultaneous removal of all examined metal ions from water solutions. Particles with EDTA functionalization were found to be highly selective for Cu and Cr removal, while PDCA functionalization of nZVI shows selective and efficient adsorption of Cu, Cr and V in aqueous medium. The particles functionalized by using different molar ratio of iron and EDTA ligand have demonstrated the similar metal ions removal efficiency. By contrast, functionalization of iron nanoparticles with PDCA by both used ratios showed a more efficient metal ions adsorption in the case when the smaller ellipsoidal particles occurred.

## Conflicts of interest

There are no conflicts to declare.

## Supplementary Material
